# Central Nervous System Lymphoma: The Great Mimicker—A Single-Institution Retrospective Study

**DOI:** 10.1155/2023/8815502

**Published:** 2023-05-16

**Authors:** Danielle A. Bazer, Ewa Zabrocka, Nicholas Koroneos, Agnieszka Kowalska

**Affiliations:** ^1^Department of Neurology, Stony Brook University Hospital, Health Sciences Tower T12/020, Stony Brook, New York 11794-8121, USA; ^2^Department of Neurology, Johns Hopkins University, 201 N Broadway, 9th Floor Mailbox #3, Baltimore, MD 21287, USA; ^3^Department of Radiation Oncology, Stony Brook University Hospital, Level 2, Stony Brook, New York 11794-8121, USA

## Abstract

**Background:**

Primary central nervous system lymphoma (PCNSL) is a rare, aggressive form of non-Hodgkin lymphoma contained in the brain and the spinal cord as well as the meninges, cranial nerves, eyes, and cerebrospinal fluid (CSF). Due to its variable presentation and lack of associated B-symptoms, it is quite challenging to diagnose PCNSL, if there is not a high level of suspicion.

**Methods:**

This is a retrospective case series examining 13 human immunodeficiency virus- (HIV-) negative patients with PCNSL and DLBCL type, with a median age of 75 years old.

**Results:**

The most common presenting symptom was altered mental status. The frontal lobes, basal ganglia, cerebellum, and corpus callosum were most affected. Prior to brain biopsy, 4/13 patients were on steroids, which did not affect biopsy results and the average time to diagnosis was 1 month. 9/13 patients who did not receive steroids had an average time to diagnosis of less than 1 month.

**Conclusion:**

Although steroid administration did not appear to diminish the yield of the biopsy, it is a best practice to withhold steroids prior to biopsy to decrease the time to diagnose PCNSL.

## 1. Introduction

Primary central nervous system lymphoma (PCNSL) is a rare, aggressive form of non-Hodgkin lymphoma contained in the brain, meninges, cranial nerves, eyes, cerebrospinal fluid (CSF), and the spinal cord [1, 2]. The majority of the cases of PCNSL are diffuse large B-cell lymphoma (DLBCL), estimating 90-95% of cases [1–3]. Individuals older than 60 years old are preferentially at risk for PCNSL, as the incidence is 0.5 per 100,000 individuals [4, 5].

Typical presenting symptoms are neurological in etiology, rather than classical “B” symptoms of fever, weight loss, and night sweats [4]. Half of all patients present with cognitive and behavior changes [3].

It is standard to have evaluation of the brain with magnetic resonance imaging (MRI) with contrast [4]. MRIs will typically reveal homogenously enhancing, often singular, lesion located supratentorially with preference of the cerebral hemispheres, corpus callosum, basal ganglia, and thalamus. Due to this predilection of deep structures, seizures are rarely associated with PCNSL [1, 3, 4].

The diagnosis is made by stereotactic biopsy or flow cytometry of the CSF lymphocytes [2, 6]. In 10-30% of patients, lymphoma cells are detected in the CSF [7]. Key markers are CD19, CD20, CD78a, CD10, B-cell CLL/lymphoma2 (BCL-2), B-cell CLL/lymphoma 6 (BCL-6), melanoma-associated antigen mutated 1 (MUM-1), and interferon regulatory factor 4 (IRF4) [3].

Many patients are given steroids prior to biopsy to reduce symptoms, as the disease is highly responsive to steroids. However, it is recommended to hold off on administering steroids until after the biopsy is completed, as the intervention will likely diminish the yield of the stereotactic biopsy as well as increase the severity of complications with treatment [2, 3, 6].

Our case series is aimed at reviewing the myriad of presentations of PCNSL and reviewing the diagnostic challenges that are encountered as a result of the variability. Although this disease is rare and complicated to diagnose, a small case series still retains value.

## 2. Materials and Methods

This is a retrospective case series examining human immunodeficiency virus- (HIV-) negative patients with PCNSL and DLBCL type, at our university hospital from the years 2010-2020. The data was obtained from the chart review. The study was approved by the Internal Review Board for publication. Inclusion criteria were PCNSL and DLBCL, in patients greater than 18 years old from the years 2010-2020. It was required that the patients have their initial MRI, work-up, and treatment plan established at our institution. We excluded individuals who had their initial treatment and/or work-up at an outside institution, as well as HIV-positive patients. We also excluded patients with an active concurrent malignancy diagnosis requiring treatment.

## 3. Results

Our series contains 13 patients, 8/13 of which were female and 5/13 were male. The average age of presentation was 69.62 (range: 51-80 years old, median age: 75). 11/13 patients were Caucasian, and the remaining 2/13 patients were Latino. Two patients had a previous, remote, nonhematologic cancer diagnosis with treatment and were deemed in remission. All patients (13/13) were HIV negative.

The median time from initial symptoms to final diagnosis was 1 month (range of same month to 6 months). Six out of thirteen patients were diagnosed within the same month, as presentation of initial symptoms. If the patients were given steroids prior to lumbar puncture (LP) or brain biopsy, the average time to diagnosis was 1 month, whereas without steroids, the time to diagnosis was less than 1 month. 11/13 patients had a neurology consultation upon initial presentation. The presenting constellation of symptoms predominantly included altered mental status (6/11), weakness (4/11), dizziness (2/11), and seizure-like activity (1/11). Seizure-like activity is defined in this context as convulsions, jerking, and starring spells.

**Figure 1:**
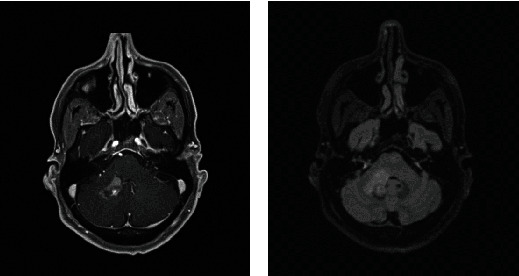
Masslike T2/FLAIR hyperintense enhancing lesion centered in the right brachium pontis extending into the dorsal pons and medulla with extension to the right trigeminal nerve root entry zone with additional multiple nonenhancing white matter lesions in the supratentorial and infratentorial brain. Radiographic differential diagnosis: tumefactive demyelination, primary CNS neoplasm, and leptomeningeal enhancement.

**Figure 2:**
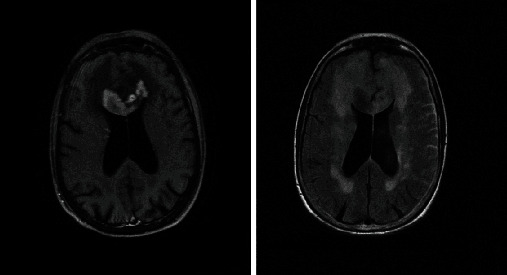
Expansion of the genu and proximal body of the corpus callosum which demonstrates heterogenous enhancement postcontrast with vasogenic edema and scattered areas of enhancement within the frontal lobes. Radiographic differential diagnosis: lymphoma, demyelinating disease, or other infiltrative process.

**Figure 3:**
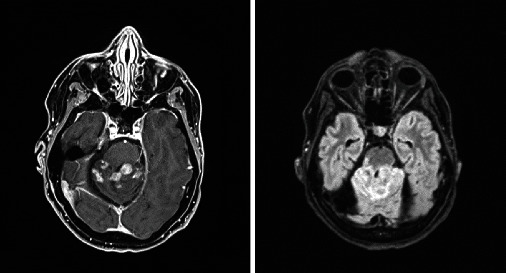
Multiple enhancing masses throughout the brain. Radiographic differential diagnosis: multiple metastatic lesions.

**Figure 4:**
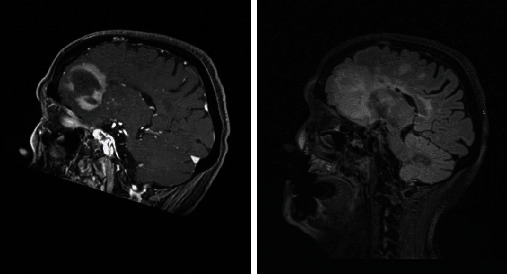
Extensive white matter changes with multiple patchy areas of intense enhancement in the left anterior and inferior frontal lobes. Radiographic differential diagnosis: PCNSL, high-grade primary neoplasm, leukoencephalopathy, and primary leukoencephalopathy with immune-reconstitution inflammatory syndrome.

**Figure 5:**
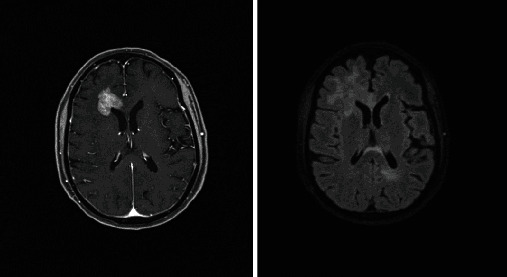
Three contrast enhancing masses in the right anterior frontal lobe wrapped around the right frontal horn, left occipital lobe, and posterior body of the corpus callosum. Radiographic differential diagnosis: PCNSL.

**Figure 6:**
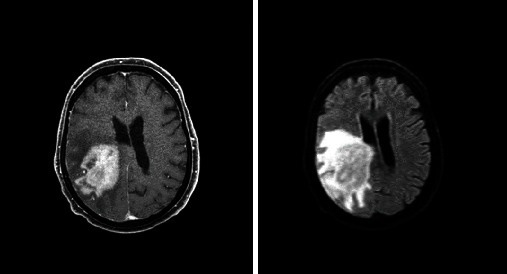
Enhancing mass in the right parietal lobe with vasogenic edema extending into the right posterior frontal lobe and right temporal lobe. Radiographic differential diagnosis: glioblastoma.

**Figure 7:**
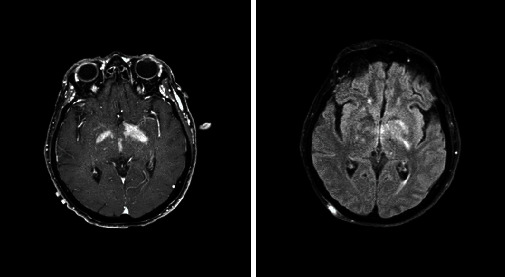
Enhancing lesions involving the bilateral cerebellar peduncles (right greater than left) as well as the bilateral internal capsules (left greater than right and the left globus pallidus). Radiographic differential diagnosis: infiltrating glioma and PCNSL.

**Figure 8:**
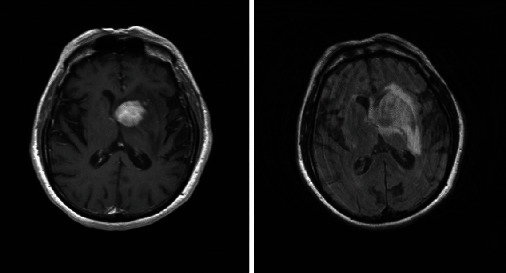
Left medial basal ganglia involving the ependyma of the left frontal horn and right temporal lobe along the course of the right temporal horn with surrounding edema. Radiographic differential diagnosis: PCNSL.

**Figure 9:**
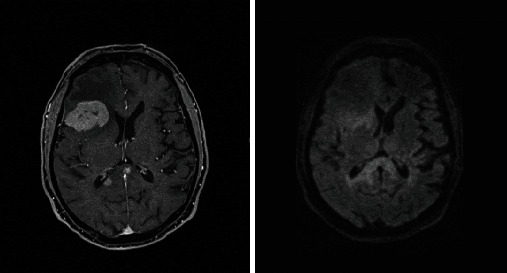
Multiple (at least 6) intra-axial brain masses with the largest in the right frontal lobe. Radiographic differential diagnosis: multifocal lymphoma and intracranial metastasis.

**Figure 10:**
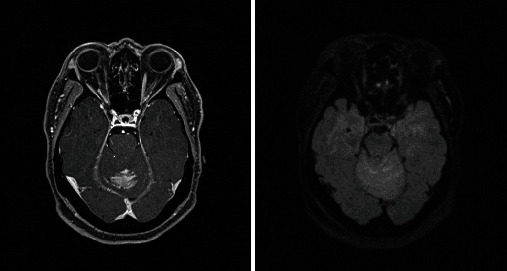
Abnormal contrast enhancing lesion superior cerebellum with surrounding vasogenic edema. Radiographic differential diagnosis: viral or bacterial cerebellitis, autoimmune inflammatory lesion, subacute infarction, and PCNSL.

**Figure 11:**
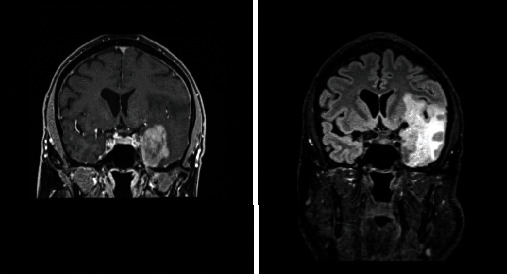
Four-centimeter extra-axial mass in the left temporal region. Radiographic differential diagnosis: meningioma.

**Figure 12:**
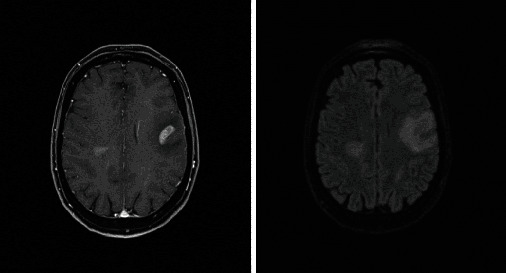
Irregular mass involving the left frontal lobe. Radiographic differential diagnosis: metastasis versus primary CNS neoplasm.

**Figure 13:**
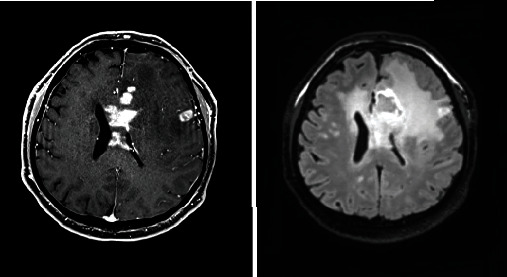
T2/FLAIR hyperintense lesion with patchy enhancement in the genu of the corpus callosum. Radiographic differential diagnosis: demyelinating lesion like ADEM and glioblastoma.

Based on the initial note, PCNSL was included in the differential diagnosis for 5/13 patients, whereas 7/13 had PCNSL included in the differential diagnosis based on the radiologist's interpretation of the presenting MRI brain. Per initial evaluation, the additional diagnoses included were metastatic disease (4/13), multiple sclerosis (2/13), glioblastoma multiforme (1/13), meningioma (1/13), encephalitis (1/13), and normal pressure hydrocephalus (1/13). The MRI was 1.5 or 3 Tesla, including the following sequences: T1 weighted, T2 weighted, proton density, diffusion weighted, and gadolinium enhanced. The patients had MRIs at the same institution; however, different machines were used throughout the course of the study. All (13/13) patients had MRI brain with and without contrast. 13/13 patients had enhancing lesions. There were 27 discrete lesions identified on all MRI scans. The predilection of the lobes affected were frontal (*n* = 5), temporal (*n* = 2), occipital (*n* = 2), and parietal (*n* = 1). Additional areas of the brain were the basal ganglia (*n* = 6), cerebellum (*n* = 4), corpus callosum (*n* = 4), and brainstem (*n* = 3) (Figures 1–13).

All (13/13) patients had a confirmatory stereotactic brain biopsy. 4/13 of the patients had steroids within the week prior to biopsy. The positive markers were as follows: CD20 (9/13), BCL-6 (9/13), Ki-67 (8/13), MUM-1 (8/13), BCL-2 (6/13), CD10 (5/13), and C-MYC (4/13).

LP was obtained in 9/13 patients prior to biopsy. LP was pursued initially as it is less invasive than a stereotactic biopsy. Biopsy was pursued in all cases for a definitive diagnosis, given the indeterminant results of the CSF analysis. A positive CSF analysis was defined using cytopathology and flow cytometry. Cytology was positive in 2/9, but 0/9 patients had positive markers in the CSF. No patients (0/9) had Epstein-Barr virus (EBV) in the CSF. Only 1/9 patients had abnormal CSF protein. LP was done once for each patient. 4/9 patients received systemic steroids prior to the LP. Three patients received dexamethasone 4 mg every 6 hours for 2-17 days, which was discontinued 3-17 days prior to the LP. One patient was treated with methylprednisolone 1,000 mg every 24 hours for 4 days, with the LP completed on the 2nd day of the treatment. Neither of the 2 patients with positive cytology result was treated with steroids prior to the LP.

**Figure 14:**
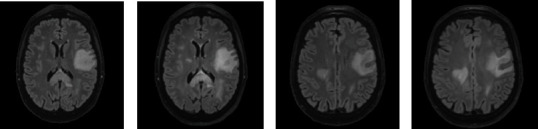
Increased FLAIR hyperintensity prior to (a, c) and 6 days after starting methylprednisolone (b, d). A new FLAIR hyperintense lesion was seen laterally to the right lateral ventricle (b).

Among the patients who received steroids prior to the LP, a follow-up imaging (brain MRI with and without contrast) was completed in one case 6 days after starting methylprednisolone and demonstrated greater FLAIR conspicuity and evidence of one new FLAIR hyperintense lesion compared with the pretreatment scan (Figure 14).

13/13 patients were eligible to receive oncologic treatment after biopsy, irrespective of age. The lifespan of the patients was 2.5 years from diagnosis to death on average for the 8/13 deceased patients, and 5/13 are alive from this cohort. 2/13 patients upon diagnosis were immediately transitioned to hospice and palliative care. The time to treatment from initial diagnosis was 18.8 days (range: 4-45 days) in the 11/13 patients who pursued treatment.

## 4. Discussion

Due to the variety of presentation of PCNSL, it is easy to lead to a misdiagnosis or delayed diagnosis, unless there is high clinical suspicion [8–10]. PCNSL should be included in the differential diagnosis, particularly when an atypical lesion is found in an older patient [4, 5, 9]. As a result, common mimickers of PCNSL should also be excluded, such as infection, demyelination, granulomatous disease, and gliomas [11]. In our case series, 5/13 patients had PCNSL included in the differential diagnosis, suggesting that the mimickers of the disease were more likely to be entertained.

Our most commonly mistaken diagnoses were metastatic disease and multiple sclerosis. In these patient populations, it can be additionally challenging to diagnose accurately, as steroids are traditionally incorporated into the treatment plan, further complicating the clinical picture and triggering further work-up of the more common entities. It may lead to a delay in diagnosis of PCNSL, as seen in our study where patients who received steroids prior to the biopsy or lumbar puncture were, on average, diagnosed with PCNSL later that those not receiving the steroids.

Although the usage of steroids may be imperative if the patient requires for symptomatic management, it is likely that the ultimate diagnosis of PCNSL will be delayed, thus delaying treatment [12]. Although it may be possible to obtain a definitive diagnosis with a patient on steroids, it is best to hold off on steroid usage (if possible) prior to biopsy [13]. In our series, the 4/13 patients who received steroids prior to brain biopsy had a positive pathology. Although this is not the optimal, it is reassuring that the biopsy results in these patients were positive. It is interesting that the 2/11patients who had abnormal cytology also received steroids prior to LP.

If there is suspicion for PCNSL, patients should be tested for HIV and EBV [9, 14, 15]. Patients who are HIV positive are at increased risk, as aggressive B-cell type form of non-Hodgkin lymphoma is the most common hematologic malignancy in HIV-positive individuals [14]. Furthermore, in HIV-positive patients, DLBCL is the most common EBV-associated lymphoma; therefore, patients should be screened for EBV in the CSF, if possible [15].

This study confirms that the key to the diagnosis of PCNSL is via histopathology from a brain biopsy. Despite PCNSL presenting quite variably, the median time from initial symptoms to final diagnosis was 1 month. Although steroids did not appear to greatly diminish the yield of biopsy in our case series, it still remains a best practice to withhold steroids prior to biopsy. The greatest limitation in this study is the small sample size; however, due to the rarity of the disease, even small studies on PCNSL may hold value in characterizing the disease.

## Data Availability

The data were obtained from chart review.
